# Reverse Genetics for Type I Feline Coronavirus Field Isolate To Study the Molecular Pathogenesis of Feline Infectious Peritonitis

**DOI:** 10.1128/mBio.01422-18

**Published:** 2018-07-31

**Authors:** Rosina Ehmann, Claudia Kristen-Burmann, Barbara Bank-Wolf, Matthias König, Christiane Herden, Torsten Hain, Heinz-Jürgen Thiel, John Ziebuhr, Gergely Tekes

**Affiliations:** a Institute of Virology, Justus Liebig University Giessen, Giessen, Germany; b Institute of Veterinary Pathology, Justus Liebig University Giessen, Giessen, Germany; c Institute of Medical Microbiology, Justus Liebig University Giessen, Giessen, Germany; d Institute of Medical Virology, Justus Liebig University Giessen, Giessen, Germany; University of California, Irvine

**Keywords:** feline coronavirus field isolates, feline infectious peritonitis, reverse genetics

## Abstract

Feline infectious peritonitis (FIP), one of the most important lethal infections of cats, is caused by feline infectious peritonitis virus (FIPV), the high-virulence biotype of feline coronaviruses (FCoVs). FIPVs are suggested to emerge from feline enteric coronaviruses (FECVs) by acquiring mutations in specific genes in the course of persistent infections. Although numerous studies identified mutations predicted to be responsible for the FECV-FIPV biotype switch, the presumed roles of specific genetic changes in FIP pathogenesis have not been confirmed experimentally. Reverse genetics systems established previously for serotype I and the less common serotype II FCoVs were based on cell culture-adapted FIPV strains which, however, were shown to be unsuitable for FIP pathogenesis studies *in vivo*. To date, systems to produce and manipulate recombinant serotype I field viruses have not been developed, mainly because these viruses cannot be grown *in vitro*. Here, we report the first reverse genetics system based on a serotype I FECV field isolate that is suitable to produce high-titer stocks of recombinant FECVs. We demonstrate that these recombinant viruses cause productive persistent infections in cats that are similar to what is observed in natural infections. The system provides an excellent tool for studying FCoVs that do not grow in standard cell culture systems and will greatly facilitate studies into the molecular pathogenesis of FIP. Importantly, the system could also be adapted for studies of other RNA viruses with large genomes whose production and characterization *in vivo* are currently hampered by the lack of *in vitro* propagation systems.

## IMPORTANCE

The availability of recombinant serotype I FCoV field isolates that are amenable to genetic manipulation is key to studying the molecular pathogenesis of FIP, especially since previous studies using cell culture-adapted FIPVs had proven unsuccessful. To our knowledge, we report the first serotype I FECV field isolate-based reverse genetics system that allows the production of high-titer recombinant virus stocks that can be used for subsequent *in vivo* studies in cats. The system represents a milestone in FCoV research. It provides an essential tool for studying the molecular pathogenesis of FIP and, more specifically, the functions of specific gene products in causing a fundamentally different progression of disease following acquisition of specific mutations. The system developed in this study will also be useful for studying other coronaviruses or more distantly related RNA viruses with large genomes for which suitable *in vitro* culture systems are not available.

## INTRODUCTION

Coronaviruses (CoVs) are positive-strand RNA viruses with genome sizes of approximately 30 kb. They are most closely related to members of the *Torovirinae*, which form a second subfamily in the family *Coronaviridae*. Together with three other families (*Arteriviridae*, *Mesoniviridae*, and *Roniviridae*), the *Coronaviridae* are part of the order *Nidovirales* (1). *Coronaviridae* are divided into four genera, *Alphacoronavirus*, *Betacoronavirus*, *Gammacoronavirus*, and *Deltacoronavirus*. Feline coronaviruses (FCoVs) are closely related to canine coronaviruses (CCoVs) and porcine transmissible gastroenteritis virus (TGEV); all three have been classified as one virus species called *Alphacoronavirus 1*. Other more distantly related viruses in the genus *Alphacoronavirus* include, for example, porcine epidemic diarrhea virus (PEDV), human coronavirus 229E (HCoV-229E), and human coronavirus NL63 (HCoV-NL63) (2).

FCoVs are widespread among cats, with seropositivity rates of 20% to 60% in the domestic cat population and up to 90% in multicat households and animal shelters (3, 4). On the basis of antigenic properties, FCoVs can be classified into two serotypes (5–7). The vast majority (80% to 95%) of natural infections occurring worldwide are caused by serotype I FCoVs, while serotype II FCoVs are less common in the field and mainly occur in Asia (3, 8–11). Interestingly, serotype II FCoVs evolve by recombination between serotype I FCoVs and CCoVs (12–15). For both FCoV serotypes, two biotypes with fundamentally different pathogenicities in cats have been defined. Feline enteric coronavirus (FECV) causes persistent infections of the gut with only minor or no clinical symptoms (16–18). In sharp contrast, feline infectious peritonitis virus (FIPV), the second biotype, causes a fatal systemic disease termed feline infectious peritonitis (FIP). It is characterized by fibrinous and/or granulomatous serositis, protein-rich serous effusion in body cavities, and granulomatous lesions in various organs (19–23).

It is now generally accepted that harmless FECVs evolve into highly pathogenic FIPVs by accumulating mutations in their genomes (24, 25). The parental FECV and the resulting FIPV from the same cat are referred to as a “virus pair.” To date, the mutations responsible for FECV-FIPV biotype switch have not been identified. However, on the basis of extensive comparative sequence analyses of FECV and FIPV isolates, it was concluded that mutations in the S and accessory genes are involved in the development of FIP (25–32). In this regard, accessory gene 3c attracted particular interest because it was found to contain deletions, frameshift mutations, and other nonsynonymous mutations in about 80% of the FIPV isolates, while FECVs carry an intact 3c gene. Accordingly, mutations in 3c were considered important virulence markers associated with FIP development (25, 32). More-recent publications suggest that an intact 3c gene is required for viral replication in the gut and that, in contrast to previous suggestions, 3c mutations are not necessarily linked to the development of FIP (26, 33–35). However, at this stage, the possibility cannot be excluded that mutations leading to truncation or loss of 3c coding sequences contribute to the emergence of viruses causing FIP. In reports of recent studies based on comparative sequence analyses of complete FECV and FIPV genomes, three substitutions in the S gene were suggested to discriminate FIPVs from FECV (26–28). Two of these changes were located in the fusion peptide (FP) and one in the heptad repeat 1 (HR1) region of the S protein. It was proposed that these substitutions change the viral cell tropism and enable efficient infection of monocytes/macrophages. Another study concentrated on differences in the furin cleavage site located between the S1 and S2 domains of the S protein (29). The respective analyses revealed that all of the FECVs contained an intact and functional furin cleavage motif whereas 10 of 11 FIPVs had amino acid changes within (or very close to) the furin cleavage site, thereby affecting the efficiency of furin-mediated S protein cleavage. Modulation of the furin cleavage by substitutions in the respective motif was proposed to be important for the switch from FECV to FIPV (29, 36).

Comprehensive comparative sequence analyses of FECVs and FIPVs led to the identification of mutations assumed to be relevant for the generation of FIPVs. However, the idea that particular mutations in the FCoV S gene and/or accessory genes indeed lead to a biotype switch from FECV to FIPV remains to be experimentally verified. In order to assess the involvement of mutations in FIPV development, the generation of well-defined viruses by reverse genetics is required (37). One major obstacle for studies on the molecular pathogenesis of FIP is the failure to grow serotype I field FECVs/FIPVs in standard cell culture systems (38). Thus far, reverse genetics systems for FECV field isolates have not been available. Only a few serotype I FIPV laboratory strains can be grown *in vitro*, but those viruses lost their ability to induce FIP due to cell culture adaptation (6, 39–42). Although serotype II FIPVs can be grown in cell culture and some of them also induce FIP, the lack of corresponding serotype II FECVs limits their use (15, 17).

In this article, we describe the establishment of the first reverse genetics system for a serotype I FECV field isolate. We show that this system overcomes the limitation caused by the inability to propagate field viruses *in vitro* and enables efficient recovery of recombinant serotype I FECVs from a cDNA clone. Importantly, we provide evidence that the recovered recombinant FECVs induce productive infection in the natural host with features resembling those of natural infections caused by these viruses.

## RESULTS

### Complete genome sequence of a serotype I FECV field isolate.

In this study, we aimed to establish a reverse genetics system based on a serotype I FECV field isolate. As starting material, fecal samples from a clinically healthy cat with a long-term history of FECV shedding were collected. The full-length genomic sequence was determined by sequence analyses of overlapping reverse transcription-PCR (RT-PCR) fragments amplified from viral RNA and was deposited in GenBank (see below). The 29,298-nucleotide (nt) genome of the serotype I FECV field isolate had the typical genome organization of FCoVs. The 5′ untranscribed regions (5′UTRs) and 3′UTRs of the FECV genome were found to comprise 311 and 276 nt, respectively. Downstream of the replicase gene (nt 312 to 20416), four structural protein genes coding for the spike protein (S) (nt 20413 to 24810), envelope protein (E) (nt 25897 to 26145), membrane protein (M) (nt 26156 to 26947), and nucleoprotein (N) (nt 26960 to 28087) are located. Accessory genes 3a, 3b, and 3c (nt 24822 to 25910) are located between the S and E genes, whereas accessory genes 7a and 7b are located downstream of the N gene (nt 28092 to 29022).

### Recovery of chimeric FECV with serotype II S gene.

Vaccinia virus-based systems have been shown to be excellent tools for the generation and genetic manipulation of full-length coronavirus (CoV) cDNA clones that can be used to produce full-length genome RNA that, following transfection, initiates a viral replication in suitable cells (40, 43–48). Generally, virus titers obtained after transfection (passage 0) are low and the production of high-titer virus stocks of the recombinant viruses requires an amplification step *in vitro*. Accordingly, reverse genetic systems have been established only for CoVs (including FCoVs) for which cell culture systems are available that efficiently support viral replication (40, 43–48). Serotype I FECV field isolates cannot be propagated in standard cell culture systems. To address this problem, we sought to recover recombinant FECV without any *in vitro* passaging steps. As a first step toward this goal, we generated a recombinant serotype I FECV in which the S gene was replaced with that of serotype II FCoV strain 79-1146. The latter virus was chosen as an S gene donor because previous experiments had demonstrated that this particular S gene conveyed efficient viral growth *in vitro* (40, 47). Using this approach, we sought to address the issue of whether the genome of the FECV field isolate characterized in this study supports the replication of and gives rise to the production of infectious FECV progeny when engineered as a chimera with the 79-1146 S gene.

**FIG 1:**
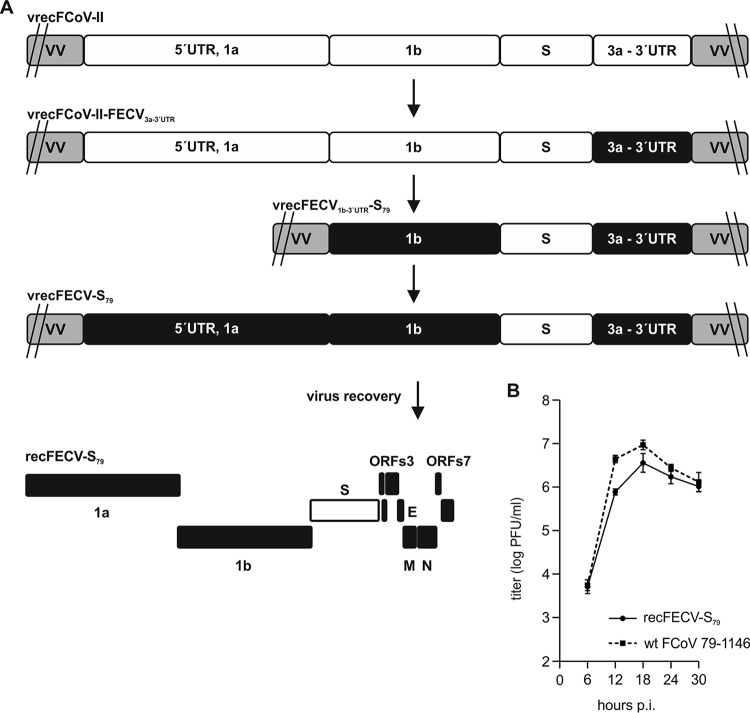
Generation of vrecFECV-S_79_ and characterization of recFECV-S_79_. (A) The strategy used to generate vrecFECV-S_79_ is presented. Recombinant vaccinia virus (VV) (vrecFCoV-II) was modified in multiple steps to replace 79-1146-derived sequences (white boxes) with the corresponding parts of the FECV field isolate (black boxes), resulting in vrecFECV-S_79_. The intermediate vaccinia viruses vrecFCoV-II-FECV_3a-3´UTR_ and vrecFECV_1b-3´UTR-_S_79_ are shown. A schematic representation of recovered recFECV-S_79_ ORFs is shown. (B) Growth kinetics of recFECV-S_79_ and serotype II FCoV strain 79-1146 after infection of FCWF cells at a multiplicity of infection (MOI) of 0.01. Results of three independent experiments are shown. wt, wild type.

On the basis of vrecFCoV-II containing the full-length cDNA of serotype II FCoV strain 79-1146 (40), we constructed recombinant vaccinia virus vrecFECV-S_79_, which contains the serotype II FCoV strain 79-1146 S sequence in the backbone of the serotype I field isolate-derived cDNA. As shown in Fig. 1A, the modification of vrecFCoV-II was carried out in multiple steps by vaccinia virus-mediated homologous recombination. First, the 79-1146-derived sequences downstream of the S gene (3a, 3b, 3c, E, M, N, 7a, 7b, and the 3′UTR) were replaced by the corresponding parts of the FECV field isolate cDNA, resulting in vrecFCoV-II-FECV_3a-3´UTR_. Second, serotype II-derived 5′UTR and open reading frame 1a (ORF1a) sequences were removed from the cDNA and the serotype II ORF1b was replaced by the corresponding FECV part. Thus, the resulting vrecFECV_1b-3’UTR_-S_79_ vaccinia virus contained the FECV-derived ORF1b, ORF3abc, E, M, N, 7ab, and 3′UTR sequences and the S gene of serotype II FCoV strain 79-1146. Finally, to generate vrecFECV-S_79_, the missing parts (5′UTR and ORF1a) of the FECV field isolate were introduced into this cDNA construct. The sequence of the full-length FCoV cDNA inserted into the vaccinia virus genome was verified by sequence analysis.

To recover recombinant FECV with serotype II S protein (recFECV-S_79_), vaccinia virus DNA derived from vrecFECV-S_79_ was prepared, cleaved with ClaI restriction enzyme, and used as a template for *in vitro* transcription as described previously (46). The *in vitro*-transcribed RNA was electroporated into BHK cells, which express the homologous FECV N protein (BHK-Tet/ON-FECV-N). After 24 h of incubation, the supernatant of electroporated cells was harvested. Upon infection of feline cells with recFECV-S_79_-containing supernatant, a cytopathic effect (CPE) developed. The identity of recFECV-S_79_ was confirmed by sequence analysis of RT-PCR products generated from viral RNA of infected Felis catus whole-fetus (FCWF) cells. The recombinant virus displayed growth characteristics similar to those displayed by serotype II FCoV strain 79-1146 and reached peak titers of 6 × 10^6^ PFU/ml at 18 h postinfection (p.i.) (Fig. 1B). The plaque morphology of the recombinant virus was indistinguishable from that of serotype II FCoV strain 79-1146 in FCWF cells (data not shown).

Taking the results together, we were able to generate a FCoV clone that contained a serotype II FCoV S gene sequence in the genomic background of serotype I FECV field isolate cDNA. Successful recovery and *in vitro* cultivation of recombinant viruses (recFECV-S_79_) in feline cells led us to conclude that the serotype I FECV field isolate-derived virus backbone is fully functional and enables efficient virus replication if the virus contains an S protein of an established laboratory strain.

### Generation and characterization of recombinant serotype I FECV.

**FIG 2:**
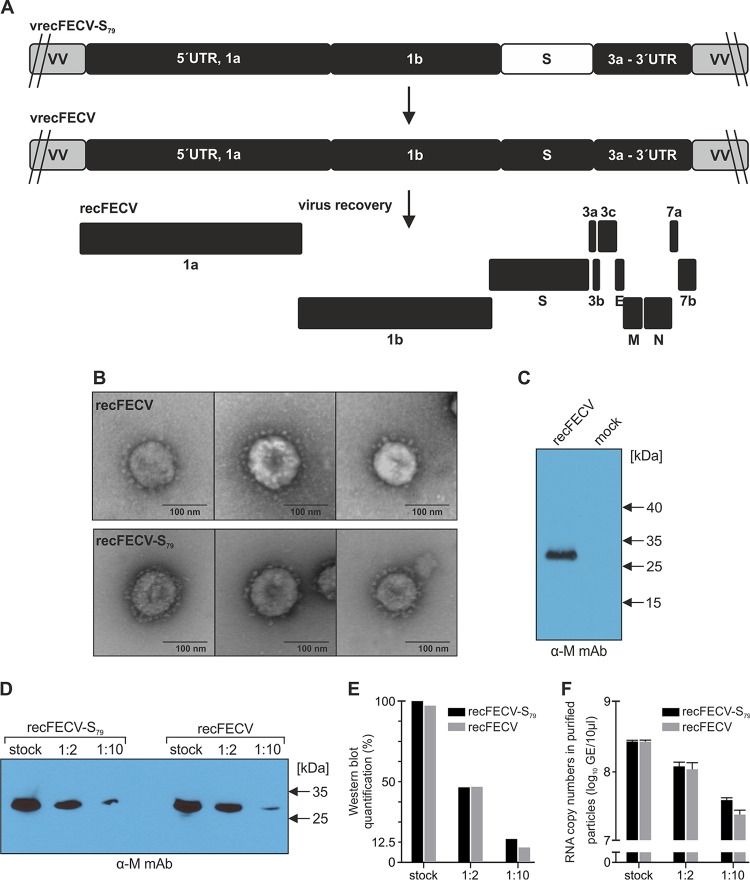
Generation and characterization of recFECV. (A) The genome organization of recombinant vaccinia virus vrecFECV and recovered recFECV is shown. (B) Electron micrographs of purified recFECV and recFECV-S_79_ originating from supernatant of electroporated cells are depicted. Negative staining was performed with 1% uranylacetate. (C) Detection of M protein in purified recFECVs using Western blot analysis with anti-M monoclonal antibody (α-M mAb). Cells were electroporated either with recFECV RNA or with PBS (mock). Supernatants were harvested 24 h after electroporation and purified by two rounds of ultracentrifugation. A 5-μl volume of purified particles was separated by SDS-PAGE (10%) under reducing conditions and analyzed by Western blotting. (D and E) Comparison of the amounts of FCoV M protein in recFECV-S_79_ versus recFECV. Ten-microliter volumes of purified viruses (stock) as well as their 1:2 and 1:10 dilutions were separated by SDS-PAGE (10%) under reducing conditions, and the results were analyzed by Western blotting using anti-M monoclonal antibody (α-M MAb) (D) and quantified (E). The intensity of the bands was analyzed using a ChemiDoc imaging system and Image Lab software. The intensity of each band was compared to that of the recFECV-S_79_ stock. (F) Ten-microliter volumes of purified viruses (recFECV and recFECV-S_79_) as well as their 1:2 and 1:10 dilutions were subjected to RNase A digestion to remove RNA outside viral particles. Upon RNA extraction, genome copy numbers in each of the fractions (stock; 1:2 and 1:10 dilutions) were determined (by qRT-PCR) and compared. GE, genome equivalents.

Our ultimate goal was the generation and recovery of a recombinant serotype I FECV without any heterologous sequences. Accordingly, the experiment performed with chimeric virus recFECV-S_79_ as described above was only an intermediate step. To generate recombinant serotype I FECV field virus with its authentic FECV S gene, the serotype II S sequence in vrecFECV-S_79_ was replaced with the appropriate S gene from the FECV field isolate, resulting in vrecFECV (Fig. 2A). The recovery of recombinant viruses from the full-length FECV cDNA was performed as described above. Since serotype I FECV cannot be propagated *in vitro*, we expected that recombinant viruses would not be able to grow in cell culture. It was therefore not surprising that inoculation of FCWF cells with the supernatant of electroporated cells did not lead to a CPE. Moreover, FECV-specific antigens could not be detected with a monoclonal antibody (anti-M) by immunofluorescence. To address the issue of whether FECV particles were released from the electroporated cells, the cell culture supernatant was subjected to an ultracentrifugation step and the pellet obtained was used for further analyses, including (i) electron microscopy, (ii) Western blotting, and (iii) RT-PCR. As shown in Fig. 2B, transmission electron microscopy studies revealed that in the supernatant of electroporated cells, virus particles with coronavirus-like morphological features, including the typical spike structures, were present which looked indistinguishable from recFECV-S_79_ particles. To verify that the recFCoV particles contained CoV structural proteins, we performed a Western blot analysis using an M protein-specific monoclonal antibody. As shown in Fig. 2C, a prominent band corresponding to the FECV structural M protein could be detected, corroborating the release of recFCoV particles from the transfected cells. Next, the incorporation of viral genome RNA into these particles was assessed by using a capsid protection assay as described previously (49). For this purpose, purified particles were subjected to RNase treatment to remove any free viral RNA prior to RNA extraction from the virus particles. The presence of viral genome RNA in these particles was confirmed by RT-PCR.

These experiments provided convincing evidence that recombinant serotype I FECVs had been recovered from the full-length cDNA clone generated in the current study. As indicated above, the titer of this virus could not be determined by plaque assay. We therefore decided to use a Western blot analysis to estimate the amount of M protein present in purified recFECV particles. As a reference, we used the M protein content of a control virus that was known to produce infectious virus progeny following electroporation of viral genome RNA and that can be titrated by virus plaque assay. Following electroporation of *in vitro*-transcribed full-length genome RNAs of recFECV and recFECV-S_79_, respectively, in a parallel experiment, cell culture supernatants of electroporated cells were collected and subjected to two rounds of ultracentrifugation. The pellet fractions were resuspended in identical volumes of NTE buffer. Equal amounts of the purified recFECV and recFECV-S_79_ stocks, along with 1:2 and 1:10 dilutions prepared from these stocks, were subjected to Western blot analysis using an M protein-specific monoclonal antibody to compare the amounts of M protein present in the viral rescue experiments performed with recFECV and recFECV-S_79_, respectively (Fig. 2D). Quantification of the M protein signals in the respective diluted and undiluted fractions revealed very similar amounts of this protein for recFECV and recFECV-S_79_ (Fig. 2E). On the basis of the comparison of the M protein contents and the titers determined for recFECV-S_79_ using a virus plaque assay with FCWF cells (5 × 10^6^ PFU/ml), we concluded that recFECV also contained about 5 × 10^6^ virus particles/ml. To further support this conclusion, we also determined the genome copy numbers in all fractions (stock and dilutions 1:2 and 1:10) of recFECV and recFECV-S_79_ by quantitative RT-PCR (qRT-PCR) after removal of possible RNA molecules outside the particles (capsid protection assay). This experiment revealed very similar genomic RNA amounts of recFECV and recFECV-S_79_ in both the undiluted and the diluted material (Fig. 2F).

Taken together, the data provide conclusive evidence that recombinant serotype I FECVs could be recovered from the cDNA clone generated in this study. While the virus particles with serotype II spike (recFECV-S_79_) enabled efficient infection of cat cells, the authentic FECV spike did not allow the virus to grow and spread in a cell monolayer *in vitro*. The lack of CPE development and FECV-specific antigens in FCWF cells inoculated with recFECV-containing cell culture supernatant suggests that the *in vitro* propagation of recFECVs critically depends on the particular S gene used to construct this virus rather than on other viral genes. Furthermore, in spite of the inability to passage recFECV *in vitro*, we showed that similarly large amounts of recFECV and recFECV-S_79_ virions could be recovered from cells in passage 0 without further amplification by viral passaging in permissive cells.

### Infection of cats with recFECV and recFECV-S_79_.

Following successful recovery, the recombinant viruses were used to assess their ability to establish infection in the natural host. FCoVs with the serotype II strain 79-1146 spike have been reported to efficiently infect cats irrespective of the virus backbone (37, 40). Accordingly, recFECV-S_79_ was used as a control. Four 5-month-old seronegative specific-pathogen-free (SPF) cats were infected with 1 × 10^7^ PFU of recFECV (cats 1 and 2) or recFECV-S_79_ (cats 3 and 4). The numbers of virions used for the cat infection experiments were determined by plaque assay for recFECV-S_79_ and estimated for recFECV as described above. To avoid cross-contamination, the two groups were housed separately. Previously, oronasal and intraperitoneal (i.p.) infection routes had generally been used for experimental infections of cats with FCoVs (16, 18, 40, 41, 50–53). To mimic the natural route of FCoV infections, one animal in each of both groups (cats 1 and 3) received the virus inoculum oronasally. The remaining two cats (cats 2 and 4) were inoculated intraperitoneally. The animals were monitored for clinical signs on a daily basis for a period of 8.5 weeks. To assess virus shedding, fecal swab samples were collected from each cat on a daily basis and analyzed by nested reverse transcription-PCR (RT-PCR). Furthermore, blood samples were collected weekly to monitor FCoV-specific antibody responses in serum samples during the course of the experiment.

**FIG 3:**
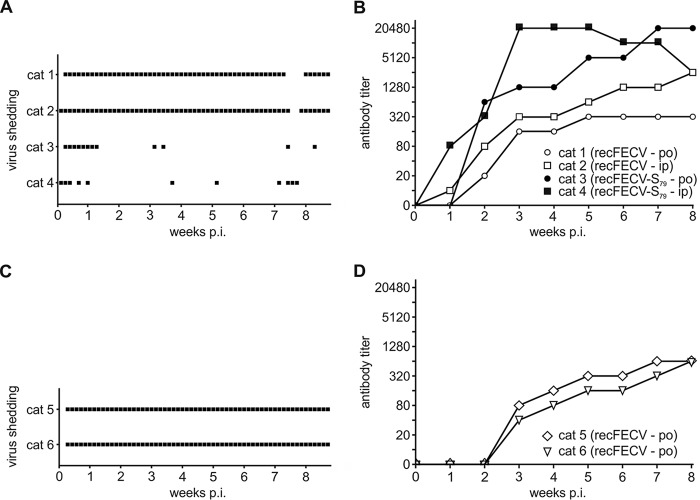
Infection of cats with recFECV and recFECV-S_79_. Groups of two SPF cats were infected with about 10^7^ particles of recFECV (cats 1 and 2) versus recFECV-S_79_ (cats 3 and 4). Cats 1 and 3 were inoculated oronasally (po), whereas cats 2 and 4 received the inoculum intraperitoneally (ip). (A) Virus shedding was monitored for cats 1 to 4 using nested RT-PCR from rectal swabs collected on a daily basis. Black boxes indicate FCoV-positive samples. (B) FCoV-specific antibody titers were determined for cats 1 to 4 weekly from serum samples by indirect immunofluorescence. In an independent second experiment, two additional animals (cats 5 and 6) were inoculated oronasally. (C and D) Virus shedding (C) and antibody titers (D) were monitored for cats 5 and 6 as described above.

After infection, all four cats developed slight fever that disappeared 3 to 4 days postinfection (p.i.). During the remaining 8 weeks, no notable clinical symptoms could be observed in any of the four cats. While FCoV-specific viral RNA was detected in fecal swab samples from the cats infected intraperitoneally (cats 2 and 4) as early as 1 day p.i., FCoV-specific viral RNA was detected 1 day later in the animals that had been inoculated via the oronasal route (cats 1 and 3). Fecal swab samples from the cats infected with recFECV (cats 1 and 2) remained positive during the entire 8.5-week period except for a few days (Fig. 3A). In contrast, fecal swab samples from the cats infected with recFECV-S_79_ (cats 3 and 4) remained FCoV positive for only about 1 week. Later, fecal samples from these cats were found to be positive only sporadically until the end of the study (Fig. 3A). To rule out the possibility that the negative results were due to the detection limit of the nested RT-PCR used in these studies, we subsequently used a qRT-PCR assay to reevaluate a total of 16 fecal samples (2 samples/week/cat) collected from cats 3 and 4. Two samples collected from cats 3 and 4 at 1 week p.i. that had tested positive by nested RT-PCR also tested positive using the qRT-PCR assay. The swabs obtained from cat 3 were found to contain 4.7 × 10^3^ and 2.9 × 10^3^ genome copies, respectively, while the swabs obtained from cat 4 contained 2.3 × 10^3^ and 1 × 10^4^ genome copies. Similarly, samples collected from cats 3 and 4 at 4 weeks p.i. that had tested positive by nested RT-PCR tested positive by qRT-PCR (3.45 × 10^2^ and 4.14 × 10^2^ genome copies for cats 3 and 4, respectively). The remaining samples collected from cats 3 and 4, respectively, that had tested negative by nested RT-PCR (week 2 to 8) remained also negative by the qRT-PCR assay. These data indicate that analyses of the results from the two assays (nested RT-PCR and qRT-PCR) led to the same conclusions.

In addition, we measured FCoV-specific antibody titers in serum samples by end point dilution using immunofluorescence on FCoV-infected Crandell Reese feline kidney (CRFK) cells (Fig. 3B). The titers against recFECV were determined using type I FCoV-infected CRFK cells, whereas the recFECV-S_79_ titers were measured using type II FCoV-infected cells. Upon infection with recFECV (cats 1 and 2), the serum antibody peak titers that developed were lower than the titers observed for the cats infected with recFECV-S_79_ (cats 3 and 4). In the cats inoculated intraperitoneally (cats 2 and 4), serum antibodies developed faster and reached peak titers earlier. After intraperitoneal infection of cat 4 with recFECV-S_79_, the serum antibody reached peak titers of >1:20,480 at 3 weeks p.i. and, after a few more weeks, declined to 1:2,560. The serum antibody titers determined for cat 3, which was infected oronasally with the same virus, reached similar peak titers (>1:20,480) but only after 7 weeks p.i. The serum antibody responses were found to be similar for the two cats infected with recFECV (cats 1 and 2), with slightly higher titers being regularly observed for cat 2, which had been infected via the intraperitoneal route. While the serum antibodies reached a peak titer of 1:2,560 in cat 2, a peak titer of 1:320 was measured for cat 1, with no further increase being recorded after week 5 p.i.

The data show that both recFECV-S_79_ and recFECV cause productive infections in cats. Note that the authentic recombinant serotype I FECV recovered from a full-length cDNA clone induced a symptomless but persistent infection in the cats that was very similar to what was seen in the cat from which this particular virus originated. Furthermore, the experiments revealed that, regardless of the route of infection, the recombinant FECVs caused persistent infections in the gut as judged by continued virus shedding in feces.

To verify that the infection of cats with recFECV can reproducibly induce a harmless persistent infection, two additional cats were infected oronasally in a separate experiment. Virus shedding and the antibody response of cats 5 and 6 were monitored for 8.5 weeks as previously described. Similarly to the first experiment, viral RNA was detected at day 2 p.i. Furthermore, all fecal swab samples collected on a daily basis tested positive throughout the course of infection (Fig. 3C). The antibody titers determined for cats 5 and 6 (Fig. 3D) were similar to the ones observed earlier for cat 1. Taken together, these data further confirm our conclusion that recFECV reproducibly causes a harmless persistent infection in cats infected via the natural route.

### Postmortem sample analyses.

**FIG 4:**
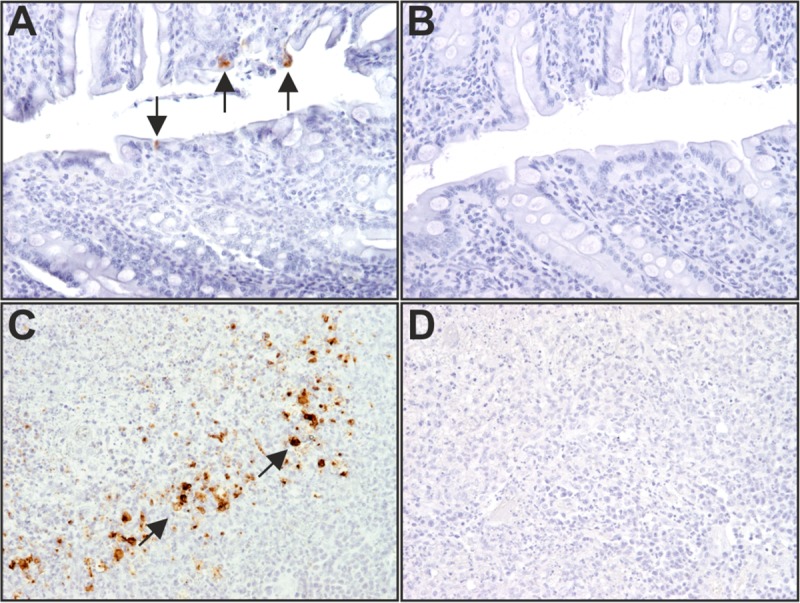
Sites of FCoV replication identified by immunohistochemistry. (A) Demonstration of FCoV-specific antigen in the colon of cat 1 using anti-M monoclonal antibody. FCoV-positive epithelial cells are marked with arrows. (B) Negative control of cat colon. Anti-M monoclonal antibody was replaced by a monoclonal antibody directed against chicken lymphocytes. (C) FCoV-specific antigen in macrophages of a lymph node from a cat naturally infected with FIPV using anti-M monoclonal antibody (α-M MAb). (D) Negative control of cat lymph node. Anti-M monoclonal antibody was replaced by a monoclonal antibody directed against chicken lymphocytes.

To conclude the animal experiment, we performed additional studies using postmortem samples obtained from cat 1, which had been infected oronasally with recFECV. To analyze virus spread *in vivo* and determine sites of viral replication, the cat was euthanized and a range of organs, including lung, liver, kidney, spleen, and abdominal lymph node samples as well as gut sections, were collected and used for subsequent analyses. Using nested RT-PCR, FCoV-specific RNA could be detected only in the colon samples. To clarify whether the negative results were due to the detection limit of the nested RT-PCR, we reinvestigated all organ samples by a qRT-PCR assay. The obtained results were in complete agreement with the outcome of the nested RT-PCR assay. As before, FCoV RNA was exclusively detected in colon samples (4.6 × 10^5^ genome copies/g tissue). Consistent with this, an immunohistochemistry (IHC) study revealed that FCoV-specific antigens could be detected only in samples originating from the colon (Fig. 4). These data are in agreement with the localization of FECV nucleic acid and proteins reported for naturally occurring persistent FECV infections (18, 54, 55) and corroborate our conclusion that the recFECV generated in this study is able to establish persistent infections that are similar to those caused by FECVs from the field.

Finally, we determined the full-length FECV genome sequence originating from cat 1 (recFECV_-cat1_). Fecal samples were collected right before the cat was euthanized and used for amplification of a set of overlapping PCR products covering the entire FECV genome. By comparing the FECV sequences derived from viral RNA isolated from fecal samples, 10 nucleotide differences from the recFECV input virus sequence were identified (Table 1). Notably, six nonsynonymous mutations were found in the S gene, two in the M gene, and one in nsp4. These data showed that the genomic sequence of the input recFECV was barely altered after 8.5 weeks (61 days) and that most of the changes were located in the S gene. Very similar results were also obtained in another study after resequencing full-length FCoV genomes several weeks postinfection (50). Desmarets et al. reported 12 nonsynonymous mutations in the FCoV genome at 84 days p.i., with 9 mutations located in the S gene, 2 in the replicase gene, and 1 in the M gene (50).

**TABLE 1:** Nucleotide and amino acid differences between recFECV and recFECV_-cat1_

Mutation	Nucleotide position in recFECV	Nucleotide in:		Protein	Amino acid substitution in the protein
		recFECV	recFECV_-cat1_		
1	7744	G	T	pp1a/pp1ab (nsp4)	G2488V
2	20612	T	C	Spike (S1)	L67P
3	20757	A	T	Spike (S1)	E115D
4	20758	A	G	Spike (S1)	T116A
5	21614	T	C	Spike (S1)	I401T
6	22121	C	A	Spike (S1)	A570E
7	23450	T	C	Spike (S2)	V1013A
8	26238	T	C	Membrane	L28P
9	26240	C	T	Membrane	L29F
10	27565	C	T	Nucleocapsid	None

## DISCUSSION

In the course of FCoV infections, which generally cause mild or no symptoms in cats, virus variants may emerge that have a fundamentally different pathogenic potential from that of the parental virus (4, 34, 38, 56–58). The biotype switch from FECV to FIPV (i.e., the lethal biotype of FCoV) has been estimated to occur in approximately 5% of persistently infected cats (4, 59). Over the past years, significant efforts by several laboratories have been made to identify genetic changes that are involved in FECV-to-FIPV biotype switches. This information is essential for understanding the molecular basis of FIP pathogenesis, which, in turn, may help the development of new diagnostic tools and, possibly, novel therapeutic and prophylactic strategies to combat this important disease. Differences between the genomes of FECVs and FIPVs have been identified by comparative sequence analyses of FECVs and FIPVs and were assumed to be responsible for the biotype switch (26–29). Although the described changes correlate with the emergence of FIPV, the specific contribution(s) of single or combined amino acid substitutions to viral pathogenesis and disease progression in infected cats was not established because suitable experimental systems were not available at the time. For this, recombinant viruses with known genome sequences, along with a set of genetically engineered mutants derived from this particular parental virus, have to be generated by reverse genetics and characterized in animal experiments. Unfortunately, FECV field isolates do not grow in cell culture and, more specifically, fail to produce infectious virus progeny unless they are adapted to *in vitro* growth conditions by serial passaging, which is known to result in mutations in different regions of the viral genome. The lack of suitable cell culture systems also posed a major obstacle in the development of reverse genetics approaches for these viruses. So far, reverse genetics systems have not been established for any of the field viruses, apart from several cell culture-adapted FCoV strains that, however, turned out to be unsuitable for studying the molecular pathogenesis of FIP (37, 40, 46, 60, 61). The major hurdle in establishing reverse genetics systems for FECV field isolates was the inability to recover infectious recombinant viruses from a cDNA clone. We therefore consider it a major achievement that an efficient FECV rescue system could be developed in the present study. Following extensive procedures performed to optimize a previously established vaccinia virus-based system (40, 44, 46, 47), high-titer recFECV virus stocks were produced.

Recombinant FECVs were generated using a two-step strategy. First, we sought to confirm that the serotype I FECV-derived backbone enables efficient viral replication if combined with the S gene of the serotype II FIPV strain 79-1146. The latter was used because this particular S protein was previously shown to support virus growth in cell culture irrespective of the FCoV backbone used (40, 47). The resulting chimeric virus, recFECV-S_79_, could be rescued and grown to high titers *in vitro*, confirming that the FECV backbone was fully functional. Second, we generated a recombinant vaccinia virus carrying the full-length FECV cDNA (including the authentic S gene). Next, BHK cells expressing the FECV N protein were electroporated with recombinant full-length recFECV RNA and supernatants collected from these cells were used to inoculate feline cells. Given that these cells are not permissive for type I field FCoVs, we were not surprised that neither CPE nor FECV-specific antigens could be detected in cat cells inoculated with the supernatant of electroporated BHK cells. However, using a range of methods, we were able to show that recFECVs had been released from the electroporated cells. The release of coronavirus-like particles was demonstrated by transmission electron microscopy, and the identity of purified (and RNase-treated) particles was corroborated further by RT-PCR analysis using FECV genome RNA-specific primers and Western blotting with a FCoV M protein-specific monoclonal antibody. The M protein content and genomic RNA content determined for the recFECV and recFECV-S_79_ preparations were found to be very similar (Fig. 2D to F), which led us to conclude that the rescue efficiencies were similar for the two viruses. The data also support the idea that the *in vitro* propagation of recombinant FECVs exclusively depends on the type of the spike protein of virus particles.

**FIG 5:**
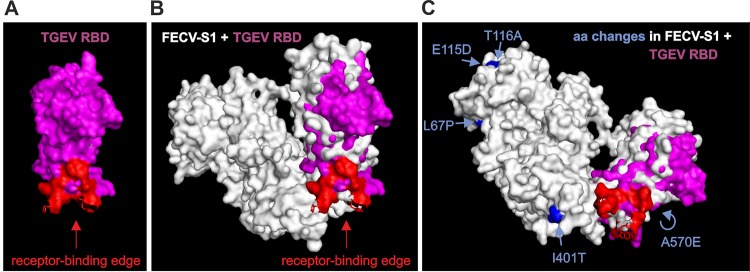
Positions of amino acid substitutions in the recFECV S protein. (A) The structure of the TGEV receptor binding domain (RBD) is presented in purple (surface view). The receptor-binding edge is shown in red. (B) The structure of the recFECV S1 subunit was modeled using Protein Homology/analogy Recognition Engine V 2.0 (Phyre 2). The surface view of the obtained recFECV S1 structure model (white) was aligned with the structure determined previously for the TGEV receptor binding domain (purple) (66) using PyMOL (PyMOL Molecular Graphics System v.1.3; Schrodinger, Inc. LLC). (C) Amino acid substitutions in the recFECV S protein are highlighted in blue. The structure was slightly rotated to show the positions of the respective amino acid (aa) substitutions in this surface representation.

Next, we addressed the issue of whether recFECV can induce a productive infection in the natural host. Cats were infected with recFECV or recFECV-S_79_ using equal amounts of virus. In the two groups, the virus was applied oronasally to one cat and intraperitoneally (i.p.) to the other. Infection with recFECV resulted in continuous virus shedding during the entire course of the experiment. Of 61 fecal swab samples obtained on a daily basis, 57 and 59 samples were found to be FCoV positive (by nested RT-PCR) in the cats infected oronasally and intraperitoneally, respectively. These data show that, irrespective of the infection route, recFECV established persistent infections of the gut. The analysis of FCoV-specific antibody responses revealed that recFECV induced relatively low antibody titers that were very similar to what was previously reported for natural infections with FECV (8, 54, 62). Similar results were observed after oronasal infection of two additional cats (cats 5 and 6) in an independent experiment, indicating that recFECV could reproducibly induce a harmless persistent infection. To determine the major sites of recFECV replication *in vivo*, the cat infected oronasally (cat 1) was subjected to a postmortem analysis at 8.5 weeks postinfection. Among the large number of tissue samples collected, only the colon tissue samples tested positive for FCoV M protein and viral genome, while viral RNA and protein could not be detected in other tissue samples, confirming that recFECV had established a persistent infection of the colon. This observation is in agreement with the localization of the virus reported for natural FECV infections (18, 54, 55). Finally, we determined the recFECV sequence from fecal samples collected right before the cat was euthanized. The sequence analysis revealed that the majority of nonsynonymous nucleotide changes mapped to the S gene and particularly to the S1 subunit. Desmarets et al. found 8 nonsynonymous mutations in the S1 subunit of the FCoV spike at 84 days p.i. (50). Since the S1 domain is known to be a major antigenic determinant for the induction of adaptive immune responses (54, 63–66), it is conceivable that the observed changes in this subunit represent immune escape variants. A structural model produced by Protein Homology/analogy Recognition Engine V 2.0 (Phyre 2) for the recFECV S1 subunit suggests that the observed amino acid substitutions in the S1 domain map to surface regions outside the receptor-binding site (Fig. 5). This localization of amino acid substitutions on the surface of the S1 subunit (including its 0 domain) strongly suggests that these changes might contribute to viral escape from the host immune system rather than increasing/adjusting the affinity to a cellular receptor as suggested in a previous study of the HCoV-229E S protein (67). Taken together, the recFECV sequence data obtained from samples collected at a late stage of infection provide independent evidence that the recFECV produced in this study is adapted to the specific ecologic niche from which it was initially isolated (i.e., feline colon). Thus, recFECV can be expected to provide an excellent tool for future studies into the biological properties of FECVs and the genetic changes required to generate the FIPV biotype.

In contrast to the data obtained for recFECV, infections with recFECV-S_79_ did not lead to continuous virus shedding. The samples tested positive (with nested RT-PCR) only sporadically (in 12 and 11 of 61 fecal swab samples) in the cats infected oronasally and intraperitoneally, respectively. Data generated by qRT-PCR confirmed these results. Interestingly, and in contrast to the titers obtained for FCoV, very high (>1:20,480) serum antibody titers were determined for both recFECV-S_79_-infected cats, reflecting a strong immune response that was most likely due to a systemic infection. The lack of clinical symptoms in the cats infected with FECV-S_79_ indicates that the S protein alone of an FIPV strain does not lead to a biotype switch even though it may be responsible for a transition from an enteric to a systemic infection. Interestingly, by the time of necropsy (8.5 weeks p.i.), none of the organ samples obtained from the cat infected oronasally with recFECV-S_79_ tested positive for FECV by PCR and IHC. It is, therefore, tempting to speculate that the strong immune response elicited by the reFECV-S_79_ infection eliminated the virus.

The efficient rescue of recFECV and its ability to cause a persistent enteric infection in cats represent a milestone in FCoV biology research because the molecular pathogenesis of FIP can now be studied by direct approaches. Using recFECV as a tool, the role of FECV-FIPV “discriminatory mutations” in the emergence of FIPVs can be directly evaluated. For example, previously proposed “discriminatory mutations” in the S protein, such as M1058L, S1060A, I1108T, and D1108Y (26–28), as well as a nonfunctional furin cleavage site (29) can be introduced into the recFECV S gene and possible effects on the pathogenicity of the resulting recombinant viruses analyzed *in vivo*. Also, using the technology established in this study, an FECV-FIPV “virus pair” obtained from a cat suffering from FIP will serve as a basis to construct a set of chimeric serotype I field viruses to identify the genes and mutations that determine an FIPV biotype. To do this, infectious clones of an FECV and the corresponding FIPV have to be produced. Systematic exchanges of genome segments from FECV with matching genome segments from FIPV will allow the mapping of the genetic changes required for turning an FECV biotype into an FIPV biotype. Obviously, the protocols developed in this study may also be useful to develop reverse genetics systems for other RNA viruses with large genomes (such as other coronaviruses and nidoviruses) for which suitable cell culture systems are not available.

## MATERIALS AND METHODS

### Cells and viruses.

Felis catus whole-fetus 4 (FCWF-4) cells and monkey kidney (CV-1) cells were purchased from the American Type Culture Collection and from the European Collection of Cell Cultures, respectively. D980R cells were a kind gift from G. L. Smith, Imperial College, London, United Kingdom. BHK-Tet/ON cells were provided by N. Tautz, University of Luebeck, Germany. All cell lines used in this study were maintained in Dulbecco’s modified Eagle’s medium (DMEM) supplemented with 10% fetal calf serum (FCS), penicillin (100 U/ml), and streptomycin (0.1 mg/ml) in 5% CO_2_ at 37°C. recFECV-S_79_ was propagated and titrated in FCWF-4 cells. Recombinant vaccinia viruses were propagated and purified as described previously (40, 46, 47).

### Full-length FECV field isolate sequence.

Fecal samples of a cat with natural FECV infection were collected. The consensus sequence of the FECV genome was determined by Sanger sequencing of cloned overlapping cDNA fragments generated with RT-PCR.

### Plasmid construction and generation of recombinant vaccinia viruses.

To generate a recombinant vaccinia virus containing the full-length cDNA of a serotype I FECV field isolate (vrecFECV), the previously produced vrecFCoV-II strain was modified (40). In total, eight rounds of vaccinia virus-mediated homologous recombination performed with Escherichia coli guanosine-phosphoribosyltransferase (GPT) as a selection marker were used to exchange the serotype II FCoV strain 79-1146 cDNA with the FECV field isolate full-length cDNA in the vaccinia virus backbone. Selection of recombinant vaccinia viruses was performed as described previously (40, 46, 47, 68–70).

Two rounds of selection were applied to exchange the region from ORF3 to the 3′UTR of serotype II FCoV strain 79-1146 with the corresponding part of the serotype I FECV field isolate, resulting in vrecFCoV-II-FECV_-3a-3´UTR_. First, to construct vaccinia virus vrecFCoV-II-_ΔORF3-3´UTR-GPT_, where the region from ORF3 to the 3′UTR was replaced with the GPT gene, plasmid pGPT-_ΔORF3-3´UTR_ was used for vaccinia virus-mediated homologous recombination with vrecFCoV-II. pGPT-_ΔORF3-3´UTR_ is based on pGPT-1 (69) and contains sequences corresponding to nucleotides (nt) 24295 to 24794 of serotype II FCoV strain 79-1146 and 500 nt of the vaccinia virus genome downstream of the FCoV cDNA upstream and downstream of the GPT gene, respectively. The recombinant GPT-containing vaccinia virus obtained after GPT-positive selection was then used for vaccinia virus-mediated homologous recombination performed with plasmid pFECV_-ORF3-3´UTR_ to generate recombinant vaccinia virus vrecFCoV-II-FECV_-3a-3´UTR_ containing the sequence from ORF3 to the 3′UTR of the serotype I FECV in the serotype II FCoV cDNA. Plasmid pFECV_-ORF3-3´UTR_ is based on pGem-T (Promega) and contains nt 24295 to 24794 of serotype II FCoV strain 79-1146 and 500 nt of the vaccinia virus vector sequence downstream of the FCoV cDNA upstream and downstream of nt 24811 to 29298 of the serotype I FECV field isolate genome, respectively.

In order to generate vrecFECV_-1b-3´UTR-_S_79_, which contains ORF1b, accessory genes 3a, 3b, and 3c, the E gene, the M gene, the N gene, accessory genes 7a and 7b, and the 3′UTR of the FECV field isolate and the S gene of type II FCoV strain 79-1146, the previously generated vaccinia virus vrecFCoV-II-FECV_-3a-3´UTR_ was used for vaccinia virus-mediated recombination with plasmid pGpt_-ΔORF1_. Plasmid pGpt_-ΔORF1_ contains sequences corresponding to 500 nt of the vaccinia virus genome upstream of the FCoV cDNA and nt 20436 to 20935 of the type II FCoV strain 79-1146 genome downstream and upstream of the GPT gene, respectively. The recombinant GPT-positive vaccinia virus obtained after GPT-positive selection was then used for vaccinia virus-mediated recombination with plasmid pFECV_-ORF1b_ to generate recombinant vaccinia virus vrecFECV_-1b-3´UTR-_S_79_ after GPT-negative selection. Plasmid pFECV_-ORF1b_ is based on pGem-T and contains 500 nt of the vaccinia virus genome upstream of the FCoV cDNA and nt 12413 to 20412 of the serotype I FECV genome followed by nt 20436 to 20935 of the type II FCoV strain 79-1146 genome.

For the generation of vrecFECV-S_79_ containing the S gene of serotype II FCoV strain 79-1146 in the serotype I FECV field isolate cDNA, vrecFECV_-1b-3´UTR-_S_79_ was used for vaccinia virus-mediated homologous recombination with plasmid pGPT-FECV_-ORF1a_. This plasmid contains sequences corresponding to 500 nt of the vaccinia virus genome upstream of the FCoV cDNA followed by nt 1 to 3500 of the FECV field isolate genome upstream of the GPT gene and nt 9500 to 12912 of the serotype I FECV field isolate genome downstream of the GPT gene. The recombinant GPT-containing vaccinia virus obtained after GPT-positive selection was then used for vaccinia virus-mediated homologous recombination with plasmid pFECV_-ORF1a_ to generate recombinant vaccinia virus vrecFECV-S_79_ after GPT-negative selection. Plasmid pFECV_ORF1a_ is based on pGem-T and contains nt 3000 to 10000 of the serotype I FECV field isolate genome.

In order to generate vrecFECV containing the full-length serotype I FECV cDNA with the authentic S gene, vrecFECV-S_79_ was used for vaccinia virus-mediated homologous recombination with plasmid pGPT_-ΔS_. This plasmid contains sequences corresponding to nt 19917 to 20416 and nt 24811 to 25210 of the serotype I FECV field isolate genome upstream and downstream of the GPT gene, respectively. The recombinant GPT-containing vaccinia virus obtained after GPT-positive selection was then used for vaccinia virus-mediated homologous recombination with plasmid pFECV_-S_ to generate recombinant vaccinia virus vrecFECV after GPT-negative selection. Plasmid pFECV-_S_ is based on pGem-T and contains nt 19917 to 25210 of the serotype I FECV field isolate genome.

### Generation of a BHK-21 cell line expressing the FECV field isolate N protein.

For the recovery of recombinant FCoVs, a BHK-21 cell line expressing the N protein of the FECV field isolate was generated (BHK-Tet/ON-N_FECV_). BHK-Tet/ON cells (kind gift from N. Tautz, University of Luebeck, Germany) were transfected with a plasmid encoding the N protein of a serotype I FECV field isolate, and single cell colonies were collected after puromycin selection (4 µg/ml). Expression of FECV field isolate N protein was determined by Western blotting 24 h after addition of doxycycline (5 µg/ml) to the culture medium.

### Rescue of recombinant FCoVs from cloned cDNA.

To recover recombinant FCoVs, DNA derived from recombinant vaccinia viruses was extracted in preparative scale, cleaved with ClaI (NEB), and used as a template for *in vitro* transcription as described previously (40, 46, 47, 68, 70). The *in vitro*-transcribed RNA was electroporated into BHK-Tet/ON-N_FECV_ cells. The supernatant of electroporated cells containing recombinant FCoVs was harvested after 24 h of incubation, and half of it purified by ultracentrifugation for further analyses. In the case of the *in vivo* experiments, the second half of the supernatant of electroporated cells was used for the infection of the cats (see below). The identity of recombinant FCoVs was determined by sequence analysis.

### Purification of recombinant FCoVs.

Half of the supernatant (5 ml) of electroporated cells containing recombinant FCoVs was centrifuged at 10,000 × *g* for 20 min to remove cell debris. Then, recombinant FCoVs were purified by two rounds of ultracentrifugation. The virus was pelleted through a 30% (wt/vol) sucrose cushion (25,000 rpm, 2 h, SW28 rotor), resuspended in NTE buffer (0.1 M NaCl, 0.01 M Tris [pH 7.4], 0.001 M EDTA), and centrifuged for a second time through a 10% (wt/vol) sucrose cushion supplemented with NTE buffer (41,000 rpm, 1 h, Ti55 rotor). The obtained highly purified virus particles were resuspended in 200 µl NTE buffer und used for further experiments.

### Electron microscopy.

FCoV particles were purified and concentrated by ultracentrifugation using a sucrose cushion that contained 2% (wt/vol) paraformaldehyde to preserve virion morphology. Material from the resuspended pellets was mounted on a Pioloform (Plano, Wetzlar), carbon coated, glow discharged using 400μ copper-rhodium grids, washed with distilled water, and stained using 1% (wt/vol) uranyl acetate. The grids were examined with a Zeiss EM 910 transmission electron microscope at 80 kV. Images were taken using a magnification-calibrated side-mounted charge-coupled-device (CCD) camera (Megaview II; SIS, Muenster, Germany) at an instrumental magnification of ×80,000.

### Detection of structural protein M in purified FCoV particles by Western blotting.

For SDS-PAGE analysis, purified virus (stock and 1:2 and 1:10 dilutions) was mixed with protein sample buffer after ultracentrifugation, heated for 5 min at 94°C, and loaded on a 10% Tricine-polyacrylamide gel (71). For Western blot analysis, proteins separated by SDS-PAGE were transferred onto a nitrocellulose membrane (GE Healthcare). After blocking, the membrane was washed with PBST (phosphate-buffered saline with 0.1% Tween 20) and incubated with primary antibody F51-1 (monoclonal mouse anti-FCoV-M [6]; kind gift from T. Hohdatsu) for 1 h at room temperature. After washing with PBST was performed, the membrane was incubated with horseradish peroxidase-conjugated goat anti-mouse antibody (Dianova) for 1 h at room temperature. Bound antibodies were visualized with chemiluminescent reagent (Western Lightning Plus-ECL; PerkinElmer). The intensity of bands corresponding to the M protein was quantified using a ChemiDoc imaging system and Image Lab software (Bio-Rad).

### Capsid protection assay.

To detect genomic RNA in purified recombinant virus particles, a capsid protection assay was performed (49). Briefly, 10 µl of purified virus (stock and 1:2 and 1:10 dilutions) was digested with 100 ng RNase A (Sigma-Aldrich) at 37°C for 30 min. The reaction mixture was supplemented with 40 U RNase inhibitor (RNaseOUT, Invitrogen) and was subjected to RNA extraction followed by qRT-PCR analysis.

### One-step real-time RT-PCR to detect viral genomic RNA.

RNA was extracted from fecal swab samples with a QIAamp viral RNA Minikit and from tissue samples with an RNeasy minikit (Qiagen). Alternatively, RNAs observed after the capsid protection assay were used for qRT-PCR. To detect viral genomic RNA, ORF1b-specific primers were designed based on the determined FECV sequence. A 10-µl PCR mixture was used per reaction and contained 2.5 µl of 4× TaqMan Fast Virus 1-Step master mix (Life Technologies, Inc.), 0.9 µl forward primer F1 (5′ AGCGTTGTACTAAGAGCGTTATGGA 3′) of a 10 µM stock solution, 0.9 µl reverse primer R1 (5′ CACATCGACCTTCCTTATACAAAAAG 3′) of a 10 µM stock solution, 0.1 µl of a 10 µM stock solution of the probe (5′ 6-carboxyfluorescein [FAM]-ATGAGCAAGTCTGTTATAAC-MGB-NFQ-3′), 3.6 µl of nuclease-free water, and 2 µl sample RNA or diluted standard RNA. The reverse transcription step (50°C for 5 min) and the enzyme activation step (95°C for 20 s) were followed by 45 cycles (3 s at 95°C and 30 s at 60°C) in a StepOnePlus Real-Time PCR Systems thermocycler (Applied Biosystems).

### RNA standards for absolute quantitation.

vrecFECV DNA was used as a template to amplify a 550-nt-long PCR product covering part of the ORF1b sequence using a forward primer containing a T7 promoter sequence at its 5′ terminus (5′ ACTGTAATACGACTCACTATAGGGCTCGACTAGAACCCTGTAATGGT 3′) and a reverse primer (5′ ACAATAGCATCACAAAACGCTACAC 3′). The PCR product was gel purified and *in vitro* transcribed with RiboMAX RNA Production System-T7 (Promega). Afterward, the *in vitro*-transcribed RNA was treated with DNase I and purified using an RNeasy minikit (Qiagen). The RNA concentration was determined with NanoVue (GE Healthcare). Ten-fold serial dilutions of the RNA were made over a range of 11 log units (10^11^ to 10^1^) to generate a standard curve (efficiency, 96.056% ± 0.79%; *R*^2^, 0.999).

### Animal experiments.

The animal experiments were done in accordance with guidelines of the Hungarian legislation on animal protection. The protocol was approved by the Pest Megyei Kormanyhivatal, Budapest (assurance numbers PE/EA/2441-6/2016 and TMF/657-12/2016). For the infection of animals with recombinant FCoVs, four specific-pathogen-free (SPF) cats were used at the age of 5 months in the first experiment and a further two cats in the second experiment. Upon arrival, the kittens were tested for FCoV seronegativity and held in a FCoV-free environment for 2 weeks prior to infection. During this time, fecal and serum samples were analyzed to ensure that the cats had not been exposed to FCoVs. Groups of two kittens were kept together for the duration of the experiment (8.5 weeks) in strictly separated rooms. One animal in each group was inoculated intraperitoneally whereas the other cat was infected oronasally with supernatant of electroporated cells (1 × 10^7^ PFU in 2 ml) in the first experiment. In the experiment that followed, the two cats were infected oronasally. Fecal swab samples were collected on a daily basis and blood samples on a weekly basis.

To monitor fecal virus shedding, swab samples were subjected to RNA extraction followed by nested RT-PCR as described previously (72) and by qRT-PCR. To determine the serum antibody titers, indirect immunofluorescence assays were performed. Briefly, CRFK cells were infected with type-I FCoV strain Black or type-II FCoV strain 79-1146 and fixed. Serum samples were added in 2-fold dilutions starting at 1:10 for both infected and noninfected cells. After incubation, the binding of primary antibodies could be detected using an anti-cat conjugate (goat anti-cat cy3) (Dianova).

After 8.5 weeks, cat 1 infected oronasally with recFECV was euthanized and tissue samples as well as fecal samples from the gut were collected. The fecal sample was used to determine the full-length genome sequence. The FECV genomic RNA was extracted, and 44 overlapping PCR products with a size of 1 to 2 kb were amplified via RT-PCR. Next-generation sequencing (NGS) of the RT-PCR products was performed by amplicon sequencing using the MiSeq platform with Reagent kit v2 (300 cycles). The cDNA library for sequencing was prepared with an Illumina Nextera XT DNA library preparation kit.

Tissue samples were subjected to RNA extraction followed by nested RT-PCR (72) and qRT-PCR as well as immunohistochemistry to identify sites of FECV replication. Coronavirus antigen was detected by immunohistochemistry, applying a standard staining protocol using primary antibody F51-1 (monoclonal mouse anti-FCoV-M [6]; kind gift from T. Hohdatsu), pretreatment with target retrieval (Agilent Technologies, Hamburg, Germany) (pH 9), secondary antibody rat anti-mouse IgG (Dianova, Hamburg, Germany), and a peroxidase-based detection system (Dianova, Hamburg, Germany). Reactions were visualized by adding 3,3′-diaminobenzidine as a chromogen (Carl Roth GmbH and Co. KG, Karlsruhe, Germany). Controls consisted either of a lymph node of a FIPV-infected cat (positive control) or of a control created by replacing the primary antibody by a monoclonal antibody directed against chicken lymphocytes (negative control).

### Accession number(s).

The full-length genomic sequence determined in this work was deposited in GenBank (accession no. MG893511).

## References

[1] de Groot R, Cowley J, Enjuanes L, Faaberg K, Perlman S, Rottier P, Snijder E, Ziebuhr J, Gorbalenya A 2012 Order Nidovirales, p 785–795. *In* King AM, Carstens EB, Lefkowitz EJ (ed), Virus taxonomy: ninth report of the International Committee on Taxonomy of Viruses. Elsevier Academic, Amsterdam, the Netherlands.

[2] de Groot R, Baker SC, Baric R, Enjuanes L, Gorbalenya AE, Holmes KV, Perlman S, Poon L, Rottier PJM, Talbot PJ, Woo PCY, Ziebuhr J 2012 Family Coronaviridae, p 806–828. *In* King AM, Carstens EB, Lefkowitz EJ (ed), Virus taxonomy: ninth report of the International Committee on Taxonomy of Viruses. Elsevier Academic, Amsterdam, the Netherlands.

[3] Hohdatsu T, Okada S, Ishizuka Y, Yamada H, Koyama H 1992 The prevalence of types I and II feline coronavirus infections in cats. J Vet Med Sci 54:557–562. doi:10.1292/jvms.54.557. 1322718

[4] Pedersen NC 2009 A review of feline infectious peritonitis virus infection: 1963–2008. J Feline Med Surg 11:225–258. doi:10.1016/j.jfms.2008.09.008. 19254859 PMC7129802

[5] Hohdatsu T, Okada S, Koyama H 1991 Characterization of monoclonal-antibodies against feline infectious peritonitis virus type-II and antigenic relationship between feline, porcine, and canine coronaviruses. Arch Virol 117:85–95. doi:10.1007/BF01310494. 1706593 PMC7086586

[6] Hohdatsu T, Sasamoto T, Okada S, Koyama H 1991 Antigenic analysis of feline coronaviruses with monoclonal antibodies (MAbs): preparation of MAbs which discriminate between FIPV strain 79-1146 and FECV strain 79-1683. Vet Microbiol 28:13–24. doi:10.1016/0378-1135(91)90096-X. 1653482 PMC7117509

[7] Pedersen NC, Black JW, Boyle JF, Evermann JF, McKeirnan AJ, Ott RL 1984 Pathogenic differences between various feline coronavirus isolates. Adv Exp Med Biol 173:365–380. doi:10.1007/978-1-4615-9373-7_36. 6331125

[8] Addie DD, Schaap IA, Nicolson L, Jarrett O 2003 Persistence and transmission of natural type I feline coronavirus infection. J Gen Virol 84:2735–2744. doi:10.1099/vir.0.19129-0. 13679608

[9] Amer A, Siti Suri A, Abdul Rahman O, Mohd HB, Faruku B, Saeed S, Tengku Azmi TI 2012 Isolation and molecular characterization of type I and type II feline coronavirus in Malaysia. Virol J 9:278. doi:10.1186/1743-422X-9-278. 23171743 PMC3568730

[10] Benetka V, Kübber-Heiss A, Kolodziejek J, Nowotny N, Hofmann-Parisot M, Möstl K 2004 Prevalence of feline coronavirus types I and II in cats with histopathologically verified feline infectious peritonitis. Vet Microbiol 99:31–42. doi:10.1016/j.vetmic.2003.07.010. 15019109 PMC7117137

[11] Kummrow M, Meli ML, Haessig M, Goenczi E, Poland A, Pedersen NC, Hofmann-Lehmann R, Lutz H 2005 Feline coronavirus serotypes 1 and 2: seroprevalence and association with disease in Switzerland. Clin Diagn Lab Immunol 12:1209–1215. doi:10.1128/CDLI.12.10.1209-1215.2005. 16210485 PMC1247821

[12] Decaro N, Buonavoglia C 2008 An update on canine coronaviruses: viral evolution and pathobiology. Vet Microbiol 132:221–234. doi:10.1016/j.vetmic.2008.06.007. 18635322 PMC7117484

[13] Herrewegh AA, Smeenk I, Horzinek MC, Rottier PJ, de Groot RJ 1998 Feline coronavirus type II strains 79-1683 and 79-1146 originate from a double recombination between feline coronavirus type I and canine coronavirus. J Virol 72:4508–4514. 9557750 10.1128/jvi.72.5.4508-4514.1998 PMC109693

[14] Lin CN, Chang RY, Su BL, Chueh LL 2013 Full genome analysis of a novel type II feline coronavirus NTU156. Virus Genes 46:316–322. doi:10.1007/s11262-012-0864-0. 23239278 PMC7089305

[15] Terada Y, Matsui N, Noguchi K, Kuwata R, Shimoda H, Soma T, Mochizuki M, Maeda K 2014 Emergence of pathogenic coronaviruses in cats by homologous recombination between feline and canine coronaviruses. PLoS One 9:e106534. doi:10.1371/journal.pone.0106534. 25180686 PMC4152292

[16] Pedersen NC, Boyle JF, Floyd K, Fudge A, Barker J 1981 An enteric coronavirus infection of cats and its relationship to feline infectious peritonitis. Am J Vet Res 42:368–377. 6267960

[17] Pedersen NC, Allen CE, Lyons LA 2008 Pathogenesis of feline enteric coronavirus infection. J Feline Med Surg 10:529–541. doi:10.1016/j.jfms.2008.02.006. 18538604 PMC7130060

[18] Vogel L, Van der Lubben M, te Lintelo EG, Bekker CP, Geerts T, Schuijff LS, Grinwis GC, Egberink HF, Rottier PJ 2010 Pathogenic characteristics of persistent feline enteric coronavirus infection in cats. Vet Res 41:71. doi:10.1051/vetres/2010043. 20663472 PMC2939696

[19] Hayashi T, Goto N, Takahashi R, Fujiwara K 1977 Systemic vascular lesions in feline infectious peritonitis. Nihon Juigaku Zasshi 39:365–377. doi:10.1292/jvms1939.39.365. 916470

[20] Kipar A, Bellmann S, Kremendahl J, Köhler K, Reinacher M 1998 Cellular composition, coronavirus antigen expression and production of specific antibodies in lesions in feline infectious peritonitis. Vet Immunol Immunopathol 65:243–257. doi:10.1016/S0165-2427(98)00158-5. 9839877 PMC7119884

[21] Kipar A, May H, Menger S, Weber M, Leukert W, Reinacher M 2005 Morphologic features and development of granulomatous vasculitis in feline infectious peritonitis. Vet Pathol 42:321–330. doi:10.1354/vp.42-3-321. 15872378

[22] Pedersen NC 1987 Virologic and immunologic aspects of feline infectious peritonitis virus infection. Adv Exp Med Biol 218:529–550. doi:10.1007/978-1-4684-1280-2_69. 2829567

[23] Weiss RC, Scott FW 1981 Pathogenesis of feline infectious peritonitis: pathologic changes and immunofluorescence. Am J Vet Res 42:2036–2048. 6280518

[24] Poland AM, Vennema H, Foley JE, Pedersen NC 1996 Two related strains of feline infectious peritonitis virus isolated from immunocompromised cats infected with a feline enteric coronavirus. J Clin Microbiol 34:3180–3184. 8940468 10.1128/jcm.34.12.3180-3184.1996 PMC229479

[25] Vennema H, Poland A, Foley J, Pedersen NC 1998 Feline infectious peritonitis viruses arise by mutation from endemic feline enteric coronaviruses. Virology 243:150–157. doi:10.1006/viro.1998.9045. 9527924 PMC7131759

[26] Bank-Wolf BR, Stallkamp I, Wiese S, Moritz A, Tekes G, Thiel HJ 2014 Mutations of 3c and spike protein genes correlate with the occurrence of feline infectious peritonitis. Vet Microbiol 173:177–188. doi:10.1016/j.vetmic.2014.07.020. 25150756 PMC7117521

[27] Chang HW, Egberink HF, Halpin R, Spiro DJ, Rottier PJ 2012 Spike protein fusion peptide and feline coronavirus virulence. Emerg Infect Dis 18:1089–1095. doi:10.3201/eid1807.120143. 22709821 PMC3376813

[28] Lewis CS, Porter E, Matthews D, Kipar A, Tasker S, Helps CR, Siddell SG 2015 Genotyping coronaviruses associated with feline infectious peritonitis. J Gen Virol 96:1358–1368. doi:10.1099/vir.0.000084. 25667330 PMC4635486

[29] Licitra BN, Millet JK, Regan AD, Hamilton BS, Rinaldi VD, Duhamel GE, Whittaker GR 2013 Mutation in spike protein cleavage site and pathogenesis of feline coronavirus. Emerg Infect Dis 19:1066–1073. doi:10.3201/eid1907.121094. 23763835 PMC3713968

[30] Kennedy M, Boedeker N, Gibbs P, Kania S 2001 Deletions in the 7a ORF of feline coronavirus associated with an epidemic of feline infectious peritonitis. Vet Microbiol 81:227–234. doi:10.1016/S0378-1135(01)00354-6. 11390106 PMC7117145

[31] Herrewegh AA, Vennema H, Horzinek MC, Rottier PJ, de Groot RJ 1995 The molecular genetics of feline coronaviruses: comparative sequence analysis of the ORF7a/7b transcription unit of different biotypes. Virology 212:622–631. doi:10.1006/viro.1995.1520. 7571432 PMC7131361

[32] Pedersen NC, Liu H, Dodd KA, Pesavento PA 2009 Significance of coronavirus mutants in feces and diseased tissues of cats suffering from feline infectious peritonitis. Viruses 1:166–184. doi:10.3390/v1020166. 21994544 PMC3185486

[33] Chang HW, de Groot RJ, Egberink HF, Rottier PJ 2010 Feline infectious peritonitis: insights into feline coronavirus pathobiogenesis and epidemiology based on genetic analysis of the viral 3c gene. J Gen Virol 91:415–420. doi:10.1099/vir.0.016485-0. 19889934

[34] Chang HW, Egberink HF, Rottier PJ 2011 Sequence analysis of feline coronaviruses and the circulating virulent/avirulent theory. Emerg Infect Dis 17:744–746. doi:10.3201/eid1706.102027. 21470478 PMC3377428

[35] Pedersen NC, Liu H, Scarlett J, Leutenegger CM, Golovko L, Kennedy H, Kamal FM 2012 Feline infectious peritonitis: role of the feline coronavirus 3c gene in intestinal tropism and pathogenicity based upon isolates from resident and adopted shelter cats. Virus Res 165:17–28. doi:10.1016/j.virusres.2011.12.020. 22280883 PMC7114484

[36] Jaimes JA, Whittaker GR 2018 Feline coronavirus: insights into viral pathogenesis based on the spike protein structure and function. Virology 517:108–121. doi:10.1016/j.virol.2017.12.027. 29329682 PMC7112122

[37] Thiel V, Thiel HJ, Tekes G 2014 Tackling feline infectious peritonitis via reverse genetics. Bioengineered 5:396–400. doi:10.4161/bioe.32133. 25482087 PMC4601228

[38] Tekes G, Thiel HJ 2016 Feline coronaviruses: pathogenesis of feline infectious peritonitis. Adv Virus Res 96:193–218. doi:10.1016/bs.aivir.2016.08.002. 27712624 PMC7112361

[39] Black JW 1980 Recovery and in vitro cultivation of a coronavirus from laboratory-induced cases of feline infectious peritonitis (FIP). Vet Med Small Anim Clin 75:811–814. 6247809

[40] Tekes G, Spies D, Bank-Wolf B, Thiel V, Thiel HJ 2012 A reverse genetics approach to study feline infectious peritonitis. J Virol 86:6994–6998. doi:10.1128/JVI.00023-12. 22491466 PMC3393577

[41] Pedersen NC, Boyle JF, Floyd K 1981 Infection studies in kittens, using feline infectious peritonitis virus propagated in cell culture. Am J Vet Res 42:363–367. 6267959

[42] Pedersen NC, Black JW 1983 Attempted immunization of cats against feline infectious peritonitis, using avirulent live virus or sublethal amounts of virulent virus. Am J Vet Res 44:229–234. 6299143

[43] Casais R, Thiel V, Siddell SG, Cavanagh D, Britton P 2001 Reverse genetics system for the avian coronavirus infectious bronchitis virus. J Virol 75:12359–12369. doi:10.1128/JVI.75.24.12359-12369.2001. 11711626 PMC116132

[44] Thiel V, Herold J, Schelle B, Siddell SG 2001 Infectious RNA transcribed in vitro from a cDNA copy of the human coronavirus genome cloned in vaccinia virus. J Gen Virol 82:1273–1281. doi:10.1099/0022-1317-82-6-1273. 11369870

[45] Coley SE, Lavi E, Sawicki SG, Fu L, Schelle B, Karl N, Siddell SG, Thiel V 2005 Recombinant mouse hepatitis virus strain A59 from cloned, full-length cDNA replicates to high titers in vitro and is fully pathogenic in vivo. J Virol 79:3097–3106. doi:10.1128/JVI.79.5.3097-3106.2005. 15709029 PMC548458

[46] Tekes G, Hofmann-Lehmann R, Stallkamp I, Thiel V, Thiel HJ 2008 Genome organization and reverse genetic analysis of a type I feline coronavirus. J Virol 82:1851–1859. doi:10.1128/JVI.02339-07. 18077720 PMC2258703

[47] Tekes G, Hofmann-Lehmann R, Bank-Wolf B, Maier R, Thiel HJ, Thiel V 2010 Chimeric feline coronaviruses that encode type II spike protein on type I genetic background display accelerated viral growth and altered receptor usage. J Virol 84:1326–1333. doi:10.1128/JVI.01568-09. 19906918 PMC2812337

[48] van den Worm SH, Eriksson KK, Zevenhoven JC, Weber F, Züst R, Kuri T, Dijkman R, Chang G, Siddell SG, Snijder EJ, Thiel V, Davidson AD 2012 Reverse genetics of SARS-related coronavirus using vaccinia virus-based recombination. PLoS One 7:e32857. doi:10.1371/journal.pone.0032857. 22412934 PMC3296753

[49] Nuanualsuwan S, Cliver DO 2003 Capsid functions of inactivated human picornaviruses and feline calicivirus. Appl Environ Microbiol 69:350–357. doi:10.1128/AEM.69.1.350-357.2003. 12514015 PMC152381

[50] Desmarets LM, Vermeulen BL, Theuns S, Conceição-Neto N, Zeller M, Roukaerts ID, Acar DD, Olyslaegers DA, Van Ranst M, Matthijnssens J, Nauwynck HJ 2016 Experimental feline enteric coronavirus infection reveals an aberrant infection pattern and shedding of mutants with impaired infectivity in enterocyte cultures. Sci Rep 6:20022. doi:10.1038/srep20022. 26822958 PMC4731813

[51] de Groot-Mijnes JD, van Dun JM, van der Most RG, de Groot RJ 2005 Natural history of a recurrent feline coronavirus infection and the role of cellular immunity in survival and disease. J Virol 79:1036–1044. doi:10.1128/JVI.79.2.1036-1044.2005. 15613332 PMC538555

[52] Haijema BJ, Volders H, Rottier PJ 2004 Live, attenuated coronavirus vaccines through the directed deletion of group-specific genes provide protection against feline infectious peritonitis. J Virol 78:3863–3871. doi:10.1128/JVI.78.8.3863-3871.2004. 15047802 PMC374255

[53] Meli M, Kipar A, Müller C, Jenal K, Gönczi E, Borel N, Gunn-Moore D, Chalmers S, Lin F, Reinacher M, Lutz H 2004 High viral loads despite absence of clinical and pathological findings in cats experimentally infected with feline coronavirus (FCoV) type I and in naturally FCoV-infected cats. J Feline Med Surg 6:69–81. doi:10.1016/j.jfms.2003.08.007. 15123151 PMC7128724

[54] Herrewegh AA, Mähler M, Hedrich HJ, Haagmans BL, Egberink HF, Horzinek MC, Rottier PJ, de Groot RJ 1997 Persistence and evolution of feline coronavirus in a closed cat-breeding colony. Virology 234:349–363. doi:10.1006/viro.1997.8663. 9268167 PMC7130968

[55] Kipar A, Meli ML, Baptiste KE, Bowker LJ, Lutz H 2010 Sites of feline coronavirus persistence in healthy cats. J Gen Virol 91:1698–1707. doi:10.1099/vir.0.020214-0. 20237226

[56] Haijema B, Rottier P, de Groot R 2007 Feline coronaviruses: a tale of two-faced types, p 183–208. *In* Thiel V (ed), Coronaviruses—molecular and cellular biology. Caister Academic Press, Norfolk, United Kingdom.

[57] Pedersen NC 2014 An update on feline infectious peritonitis: virology and immunopathogenesis. Vet J 201:123–132. doi:10.1016/j.tvjl.2014.04.017. 24837550 PMC7110662

[58] Kipar A, Meli ML 2014 Feline infectious peritonitis: still an enigma? Vet Pathol 51:505–526. doi:10.1177/0300985814522077. 24569616

[59] Addie DD, Toth S, Murray GD, Jarrett O 1995 Risk of feline infectious peritonitis in cats naturally infected with feline coronavirus. Am J Vet Res 56:429–434. 7785816

[60] Haijema BJ, Volders H, Rottier PJ 2003 Switching species tropism: an effective way to manipulate the feline coronavirus genome. J Virol 77:4528–4538. doi:10.1128/JVI.77.8.4528-4538.2003. 12663759 PMC152114

[61] Bálint Á, Farsang A, Zádori Z, Hornyák Á, Dencso L, Almazán F, Enjuanes L, Belák S 2012 Molecular characterization of feline infectious peritonitis virus strain DF-2 and studies of the role of ORF3abc in viral cell tropism. J Virol 86:6258–6267. doi:10.1128/JVI.00189-12. 22438554 PMC3372193

[62] Pedersen NC 1976 Serologic studies of naturally occurring feline infectious peritonitis. Am J Vet Res 37:1449–1453. 793459

[63] Li C, Li W, Lucio de Esesarte E, Guo H, van den Elzen P, Aarts E, van den Born E, Rottier PJM, Bosch BJ 26 5 2017 Cell attachment domains of the porcine epidemic diarrhea virus spike protein are key targets of neutralizing antibodies. J Virol doi:10.1128/JVI.00273-17. PMC5446644 28381581

[64] Walls AC, Tortorici MA, Frenz B, Snijder J, Li W, Rey FA, DiMaio F, Bosch BJ, Veesler D 2016 Glycan shield and epitope masking of a coronavirus spike protein observed by cryo-electron microscopy. Nat Struct Mol Biol 23:899–905. doi:10.1038/nsmb.3293. 27617430 PMC5515730

[65] Kubo H, Yamada YK, Taguchi F 1994 Localization of neutralizing epitopes and the receptor-binding site within the amino-terminal 330 amino acids of the murine coronavirus spike protein. J Virol 68:5403–5410. 7520090 10.1128/jvi.68.9.5403-5410.1994 PMC236940

[66] Reguera J, Santiago C, Mudgal G, Ordoño D, Enjuanes L, Casasnovas JM 2012 Structural bases of coronavirus attachment to host aminopeptidase N and its inhibition by neutralizing antibodies. PLoS Pathog 8:e1002859. doi:10.1371/journal.ppat.1002859. 22876187 PMC3410853

[67] Wong AHM, Tomlinson ACA, Zhou D, Satkunarajah M, Chen K, Sharon C, Desforges M, Talbot PJ, Rini JM 2017 Receptor-binding loops in Alphacoronavirus adaptation and evolution. Nat Commun 8:1735. doi:10.1038/s41467-017-01706-x. 29170370 PMC5701055

[68] Eriksson KK, Makia D, Thiel V 2008 Generation of recombinant coronaviruses using vaccinia virus as the cloning vector and stable cell lines containing coronaviral replicon RNAs. Methods Mol Biol 454:237–254. doi:10.1007/978-1-59745-181-9_18. 19057873 PMC7121376

[69] Hertzig T, Scandella E, Schelle B, Ziebuhr J, Siddell SG, Ludewig B, Thiel V 2004 Rapid identification of coronavirus replicase inhibitors using a selectable replicon RNA. J Gen Virol 85:1717–1725. doi:10.1099/vir.0.80044-0. 15166457

[70] Florek D, Ehmann R, Kristen-Burmann C, Lemmermeyer T, Lochnit G, Ziebuhr J, Thiel HJ, Tekes G 2017 Identification and characterization of a Golgi retention signal in feline coronavirus accessory protein 7b. J Gen Virol 98:2017–2029. doi:10.1099/jgv.0.000879. 28758629 PMC7212014

[71] Schägger H, von Jagow G 1987 Tricine-sodium dodecyl sulfate-polyacrylamide gel electrophoresis for the separation of proteins in the range from 1 to 100 kDa. Anal Biochem 166:368–379. doi:10.1016/0003-2697(87)90587-2. 2449095

[72] Herrewegh AA, de Groot RJ, Cepica A, Egberink HF, Horzinek MC, Rottier PJ 1995 Detection of feline coronavirus RNA in feces, tissues, and body fluids of naturally infected cats by reverse transcriptase PCR. J Clin Microbiol 33:684–689. 7751377 10.1128/jcm.33.3.684-689.1995 PMC228014

